# Virtual anthropology: a preliminary test of macroscopic observation *versus* 3D surface scans and computed tomography (CT) scans

**DOI:** 10.1080/20961790.2020.1817270

**Published:** 2020-10-30

**Authors:** Claudine Abegg, Ilaria Balbo, Alejandro Dominguez, Silke Grabherr, Lorenzo Campana, Negahnaz Moghaddam

**Affiliations:** a Unit of Forensic Imaging and Anthropology, University Center of Legal Medicine Lausanne-Geneva, Lausanne, Switzerland; b Dipartimento di Scienze biologiche, Università di Bologna, Bologna, Italy; c School of Health Sciences (HESAV), University of Applied Sciences and Arts Western Switzerland (HES-SO), Delémont, Switzerland; d Swiss Human Institute of Forensic Taphonomy, Lausanne, Switzerland

**Keywords:** Forensic sciences, virtual anthropology, CT scan, 3D surface scan, forensic anthropology, cranial sutures

## Abstract

Virtual anthropology (VA) is based on applying anthropological methods currently used to analyse bones to 3D models of human remains. While great advances have been made in this endeavour in the past decade, several interrogations concerning how reliable these models are and what their proper use should be remain unanswered. In this research, a fundamental assumption of VA has been investigated: if the way we perceive and apply an anthropological method is truly similar when looking at bones macroscopically and through various 3D media. In order to answer, 10 skulls of known age and sex were scanned using a computed tomography (CT) scanner and a 3D surface scanner. Two observers separately applied a defined staging method to eight suture sites on these skulls, first looking at the bone macroscopically, then at the 3D surface scan, and finally on the CT scan. Two rounds of observation were carried out by each observer. Intra- and inter-observer error were evaluated, and two sample *t*-tests used to evaluate if the different types of medium used yielded significantly different observations. The results show a high degree of inter-observer error, and that data obtained from 3D surface scans differ from macroscopic observation (confidence level 95%, *P* ≤ 0.05). CT scans, in these settings, yielded results comparable to those obtained through macroscopic observations. These results offer many possibilities for future research, including indications on the kind of anthropological methods and anatomical landmarks that might be reliably transferable to the virtual environment. All current methods used in traditional anthropology should be tested, and if they prove unreliable, new techniques to analyse bones from virtual models should be developed.

**Key points**
- Large discrepancies between observation on dry bones and computer-generated 3D models (surface scans or CT scans) could lead to the re-evaluation of the suitability of traditional anthropological methods for application on 3D models.
- This preliminary study evaluates whether macroscopic, 3D surface scans, and CT scans viewings generate different observations.
- The results indicate that the data are not always coherent across all three media of observation.
- Explanations include the aspect given to the bone by the 3D software, differences between handling bones in real life *versus* on a computer, and level of expertise of the observers.

## Introduction

As virtual anthropology (VA) develops as a discipline, it is becoming an important part of forensic anthropology. Forensic anthropologists routinely perform computed tomography (CT) scans as part of their protocol, and 3D surface scanning offers new possibilities for the quick and accurate digitization of bones [1]. There are many advantages to having accurate 3D models available for research: the entire structure is accessible, a large number of analyses (volumetric, form, measurements, visualisation, etc.) can be performed, and these analyses can be replicated [2]. Moreover, the data can be preserved for long periods of time, and be made available for researchers worldwide to consult, making large-scale anthropological research more feasible. This new digital era also brings its share of problems. While having large VA corpuses would solve many research problems, acquiring CT scans of living or recently deceased persons and using them require deep ethical consideration, especially at a time where the constitution and use of skeletal collections is questioned [3]. The management of such virtual collections in the long-term perspective is also important. Finally, it is not yet known whether the 3D models acquired can be analysed accurately and reliably using traditional anthropological methods, or not.

In the past decade, many studies have started to investigate the potential of CT and 3D surface scans in forensic anthropology, by looking at how well traditional methods performed on virtual models or on how close to the original bone the models themselves are [4–6]. Overall, the results have been encouraging, suggesting that these 3D models are suitable for classical anthropological analyses. Our analysis inscribes itself within this line of research. The University Center of Legal Medicine Lausanne-Geneva (i.e. Centre universitaire romand de médecine légale, Lausanne-Genève, CURML) manages important amounts of forensic cases in Switzerland. A pioneer in the development of forensic imaging [7], CT and 3D surface scans are routinely performed at the CURML, and a series of projects on the use of CT and 3D surface scanning models in anthropology are ongoing [8, 9]. In the research presented here, 3D models were created using both a CT scanner and a 3D surface scanner. The aim of this research is to determine whether observations made macroscopically differ significantly from observations made on CT-generated models and/or 3D surface models, and to quantify those differences.

## Material and methods

### Subjects

Ten skulls of known sex and age were analysed (Table 1), stemming from cases processed at the CURML. Some of the skulls presented blunt force trauma or various pathologies, but were nonetheless included since the aim of the research was to evaluate the concordance of the observations made on the models, and not the accuracy of the method chosen to evaluate it. All human bone specimen used were issued from cases in which they were macerated and scanned for the purpose of forensic examination. Further examination for this research’s purpose was entirely non-invasive.

**Table 1.** The age and sex of the skulls used in this research (*N*=10).

Cranium No.	Age (years old)	Sex
1	25	Male
2	29	Male
3	78	Male
4	30	Male
5	83	Female
6	42	Male
7	16	Male
8	59	Male
9	38	Male
10	56	Male

### Suture examination

**Figure 1.**
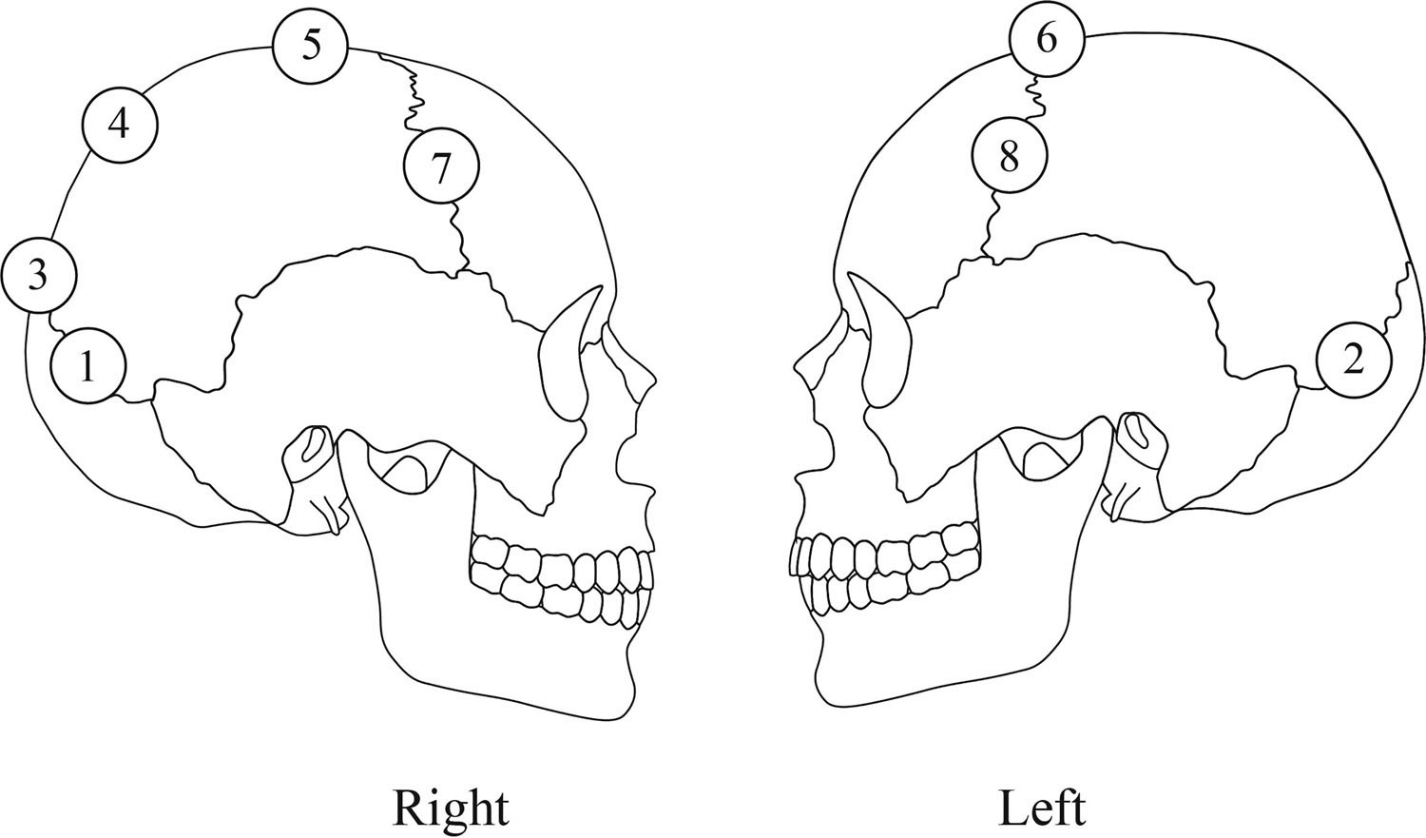
Schematic representation of the location of the eight suture points evaluated. 1: midlamboid right, 2: midlamboid left, 3: lambda, 4: obelion, 5: anterior sagittal, 6: bregma, 7: midcoronal right, 8 midcoronal left.

Eight cranial suture points were considered on every skull (Figure 1), from the method established by Meindl and Lovejoy [10] in 1985 to evaluate the age of an individual based on cranial suture closing.

The method consists in observing several cranial sutures sites and attribute a stage to each site (Table 2). According to the method, each stage for each suture point corresponds to a mean age and standard deviation. The estimated age is calculated by averaging the mean age given for each observed suture point [10]. Two observers independently graded the cranial suture points selected on the 10 skulls. Analyses were first performed macroscopically, then on the 3D surface scan models, and finally on the CT scans volume-rendering model. The process was repeated 2 weeks later, so that both intra- and inter-observer error could be evaluated. Both observers were trained in anthropological analysis, but had different levels of experience (Observer 1 was at a Masters level, Observer 2 holds a PhD in anthropology).

**Table 2.** Description of the grading system used to evaluate suture closure, adapted with permission from Meindl and Lovejoy, 1985 [10].

Suture score	Description
0	Suture observed as open, where a distinct suture line is visible
1	Suture with minimal closure (<50%)
2	Suture with significant closure (>50%), but where the suture line is still visible
3	Suture completely obliterated

### Data acquisition

Each skull was documented through photographs, using a Nikon D750 (focal length 55 mm–92 mm, aperture F/16; Melville, NY, USA). The 3D surface scans were performed through an ATOS Compact Scan 5 M rev. 01 (GOM-Gesellschaft für optische Messtechnik, Braunschweig, Germany), set with a distance of 300 mm between cameras position, and calibrated to a measuring volume of 150 mm × 110 mm × 110 mm, which leads to a point resolution of 0.062 mm. Targets (small black and white markers that the scanner uses as reference points; Φ: 1.5 mm) were placed randomly on the cranium approximately 10 cm apart to guide the scan. The mesh was created in the same software which was used for the 3D surface scanning (GOM ATOS Professional), using the “no post processing method” option, in order to preserve image resolution. Each cranium was scanned using a GE HealthCare VCT lightspeed-64 Rows (Milwaukee, WI, USA) (Table 2). CT parameters were 0.625 mm slice width, 6 mm × 0.5 mm detector-collimation, and a reconstruction increment of 0.3 mm (tube voltage: 100 kV; 120 mA; radiation dose: 12.24 mGy). Reconstructions were performed with Advantage Windows Server 3.2 (GE HealthCare) using a volume-rendering protocol (bone description) with a specular of luminous intensity set at 0.5 for observation. The upward curve was 120–600 HU (Hounsfield Unit value).

### Statistical analysis

The data were analysed using Stata (Release 16) [11]. Considering sample size and the experiment set up, a paired sample *t*-test was chosen to detect significant differences in paired data [12]. The significance level (*P*-value) for all tests was set at a base of 0.05 (95% confidence). The null hypothesis in each test was that either the two observers or the two methods scrutinised did not differ significantly in the mean age attribution. Therefore, if the *P*-value was greater than 0.05, the two set of data were not significantly different, and our null hypothesis was accepted.

## Results

All statistical test and their results (*t*-test and *P*-value) are summarised in Table 3.

**Table 3.** Summary of all *t*-tests performed during statistical analysis.

Observer No.	Parameters (Student *t*-tests)	*t*-test result (*df* = 9)	*P*-value result for the test
1	Intra-observer error, macroscopic observation	1.0638	0.3151
	Intra-observer error, 3D surface scans	0.8328	0.4265
	Intra-observer error, CT scans	1.7610	0.1121
2	Intra-observer error, macroscopic observation	3.4603**	0.0072
	Intra-observer error, 3D surface scans	0.5703	0.5825
	Intra-observer error, CT scans	0.2573	0.8027
1 and 2	Inter-observer error, macroscopic observation	2.8375*	0.0195
	Inter-observer error, 3D surface scans	3.4670**	0.0071
	Inter-observer error, CT scans	6.1696**	0.0002
	Comparing macroscopic observation and 3D surface scans	3.6861**	0.0050
	Comparing macroscopic observation and CT scans observation	1.3502	0.2099
	Comparing 3D surface scans and CT scans observations	4.5956**	0.0013
1	Comparing macroscopic observation and 3D surface scans	2.3404*	0.0440
	Comparing macroscopic observation and CT scans observation	0.4140	0.6886
	Comparing 3D surface scans and CT scans observation	4.3042**	0.0020
2	Comparing macroscopic observation and 3D surface scans	3.9339**	0.0034
	Comparing macroscopic observation and CT scans observation	1.8720	0.0940
	Comparing 3D surface scans and CT scans observation	1.0170	0.3357

*P ≤ 0.05, and therefore a confidence level of 95%.

**P ≤ 0.01, and therefore a confidence level of 99%.

### Intra-observer error

All *t*-tests comparing the first round of observations conducted by Observer 1 to the second round of observations led to the acceptance of the null hypothesis (no significant differences in data), for each of the methods used (macro, 3D, CT). Observer 2 showed significant differences in the observations when using the macroscopic method (*t*-test = 3.4603; *P* = 0.0072, Supplement Table S1) but not when using 3D surface scans (*t*-test = 0.5703; *P* = 0.5825) or CT scans (*t*-test = 0.2573; *P* = 0.8027).

### Inter-observer error

**Figure 2.**
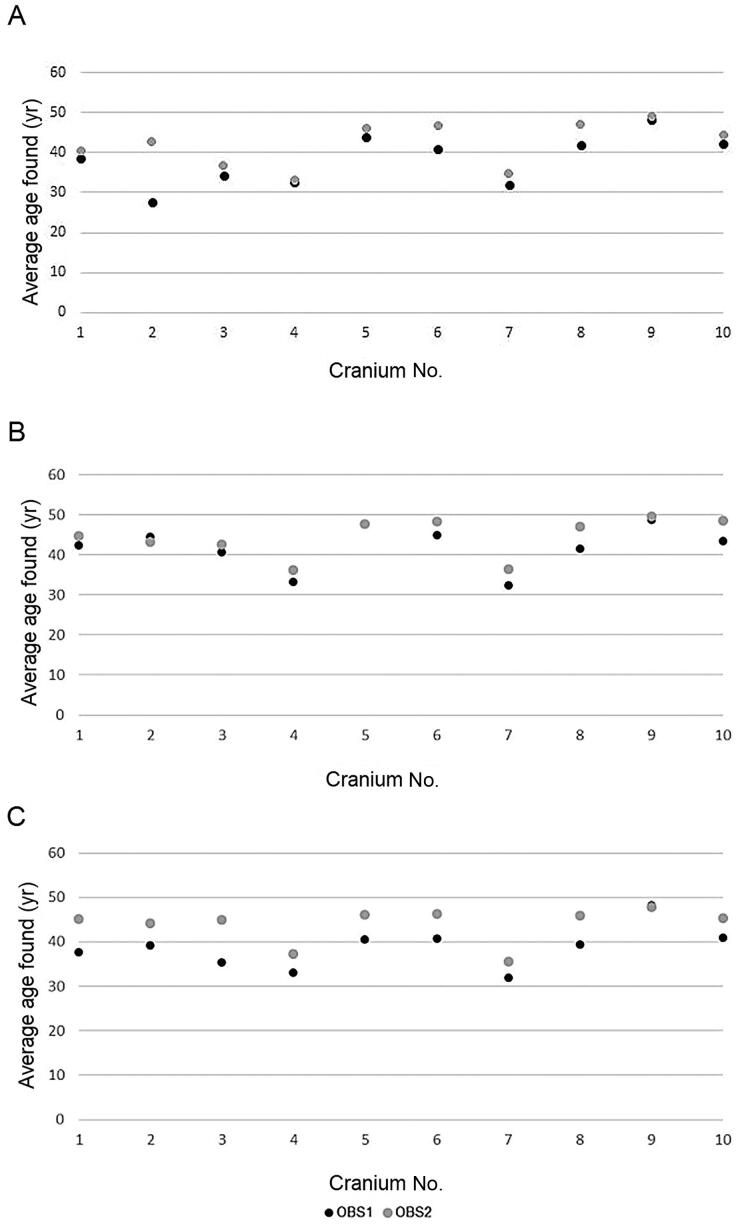
Differences in average age found by Observer 1 (OBS1) and Observer 2 (OBS2) when using macroscopic (A), 3D surface scans (B) and CT scans (C) observations.

First, comparisons of the average age given by Observer 1 and Observer 2 to each skull during their two rounds of macroscopic evaluation of the skulls was made. The null hypothesis was rejected; the observers attributed different ages to the skulls (Supplement Table S2, Figure 2A).

The same test was applied comparing Observer 1 and Observer 2’s data for 3D surface and CT scans. In each case, the null hypothesis was rejected. There were significant differences in the data obtained by each observer (Figures 2B, 2C).

### Comparing macroscopic evaluation with 3D surface scan and CT scan models

**Figure 3.**
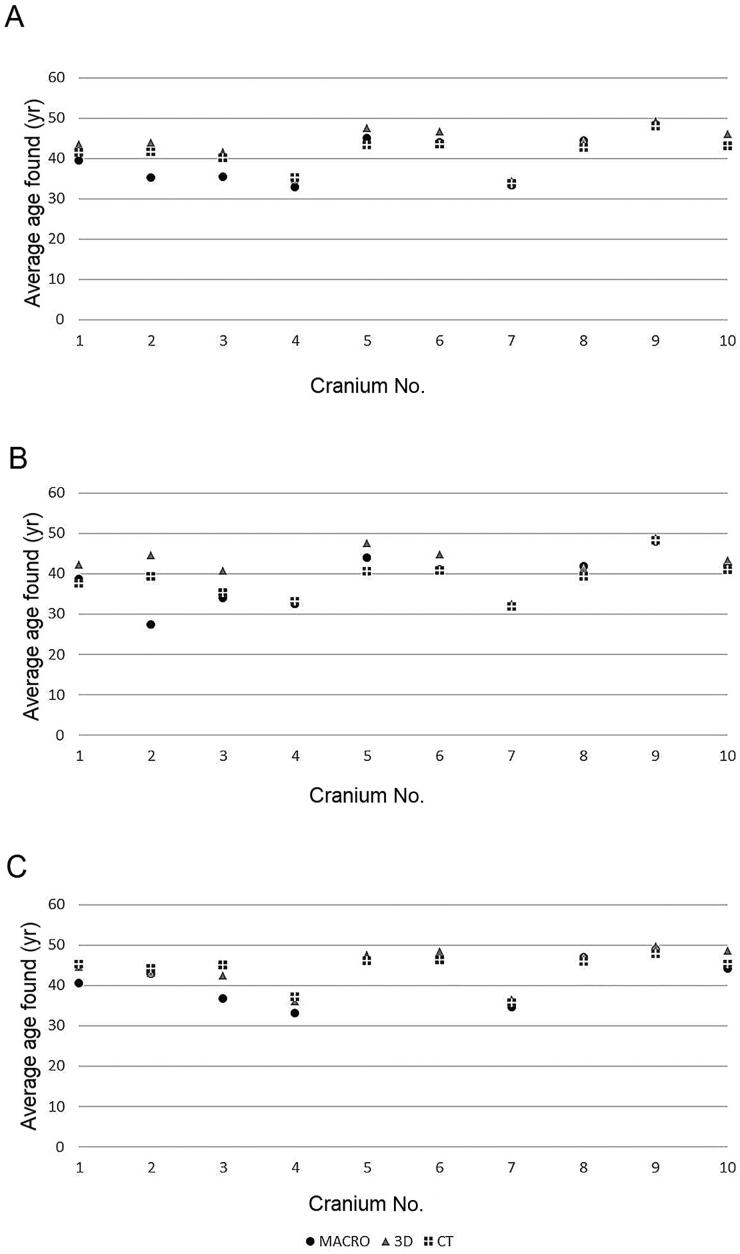
Visual representation of the differences in the observations made using all three methods, when considering the averaged data of both observers (A), Observer 1 (B), and Observer 2 (C) (this observer obtained observations that are more coherent across all three methods, despite originally having a higher degree of intra-observer error).

When comparing the results obtained through the three observation media and considering the average of the results of both observers, macroscopic observations differed significantly from those made from 3D surface scans. This holds true as well when comparing 3D surface scans and CT scans. Observing skulls macroscopically and through CT scans, however, indicated that these two methods yielded comparable results. These results are summarised in Figure 3A.

Since it was known that there was a high degree of inter-observer error, it was suspected that averaging both observers’ observations would multiply differences in observations. Observer 1 and Observer 2’s data were therefore taken into consideration separately, looking at differences between macroscopic examination of the skull, and examination of the digital models from 3D-surface scanning and CT-scanning. With Observer 1’s observations, the results were mitigated. Both “*macroscopic versus 3D surface scans*” and “*3D surface scans versus CT scans*” tests yielded significantly different results. Macroscopic observations, however, were comparable to CT scan observations (Figure 3B).

The data generated by Observer 2 yielded slightly different results. Once more, observations obtained from macroscopic and 3D surface scans were significantly different. However, when comparing macroscopic observation to CT scans, the results were similar, and the same holds true when considering 3D surface scans and CT scans. Figure 3C shows a graphical representation of these results.

## Discussion and conclusion

CT and 3D surface scanners, the most used equipment in creating VA models, have both advantages and disadvantages. CT scans allow the observation of the internal structure of the bone, which is extremely useful in both medical and anthropological examinations [4, 5, 13–16]. They have successfully been used to determine some biological parameters (sex, for example) from CT-scanned bones [17–19]. They are also expensive, and require skilled technicians to operate [1]. The post-acquisition time and the interpretation of the images obtained also necessitates some training. 3D surface scanners are quick in data acquisition and post-processing, are affordable (depending on the model used), transportable, and emit no radiation [4, 20–24]. On the other hand, they do not allow the observation of the internal structure of bones, and their accuracy depends on how skilled the person using them is, as well as the technical spectrum of the scanner and post-processing programme. All these characteristics play a role in the way they will be mobilised in future virtual anthropological research.

Before discussing the results of this study, a few observations on the method chosen to evaluate the reliability of the models in various conditions must be mentioned. Evaluating age using cranial sutures is problematic, and widely debated in anthropology, due to its inaccuracy [25–27]. The standard deviations associated with the mean ages can reach as far as ±25 years, rendering it inefficient for forensic anthropology purposes. However, the purpose of this research was not to evaluate the accuracy of the method, but to use it to see if with a predefined criterion the medium of observation (macroscopic observation, 3D surface scans, CT scans) made a difference in the results observed. Since the mean age attributed to each stage for each cranial suture point is only dependant on the decision of the observer, it can be used to evaluate whether the same stage was attributed to a given suture across all three media used.

This research yielded several quantified and qualified observations pertinent to the topic of the use of virtual models to analyse human skeletal remains. First, the method upon which the analysis was based played a role in the results obtained. It has to be pointed out that this research did not aim to prove the reliability of cranial suture closure as an aging method. Indeed, it has proven unreliable repeatedly [25–27], despite still figuring in many textbooks [28,29] although sometimes as little more than an indicator of old age [30]. We did however expect cranial sutures to look similar across all three media so that there would be no major obstacle to their observation. The criteria of the method are quite problematic. Stage 0 (suture completely open) and 3 (suture obliterated) are quite clear-cut. The remaining two criteria (1: less than 50% of the suture closed and 2: more than 50% of the suture closed) are extremely dependant on the viewing angle, lighting, and the experience of the observer. This lack of precision contributed to the first quantified result of the research: the high level of inter-observer error. Furthermore, the observers differed in their number of years of expertise handling human remains — while observers were mostly coherent within their own sets of data, it appears the method yielded different results when applied by different observers, further reinforcing the idea that the chosen method might not be ideally suited to such analyses.

**Figure 4.**
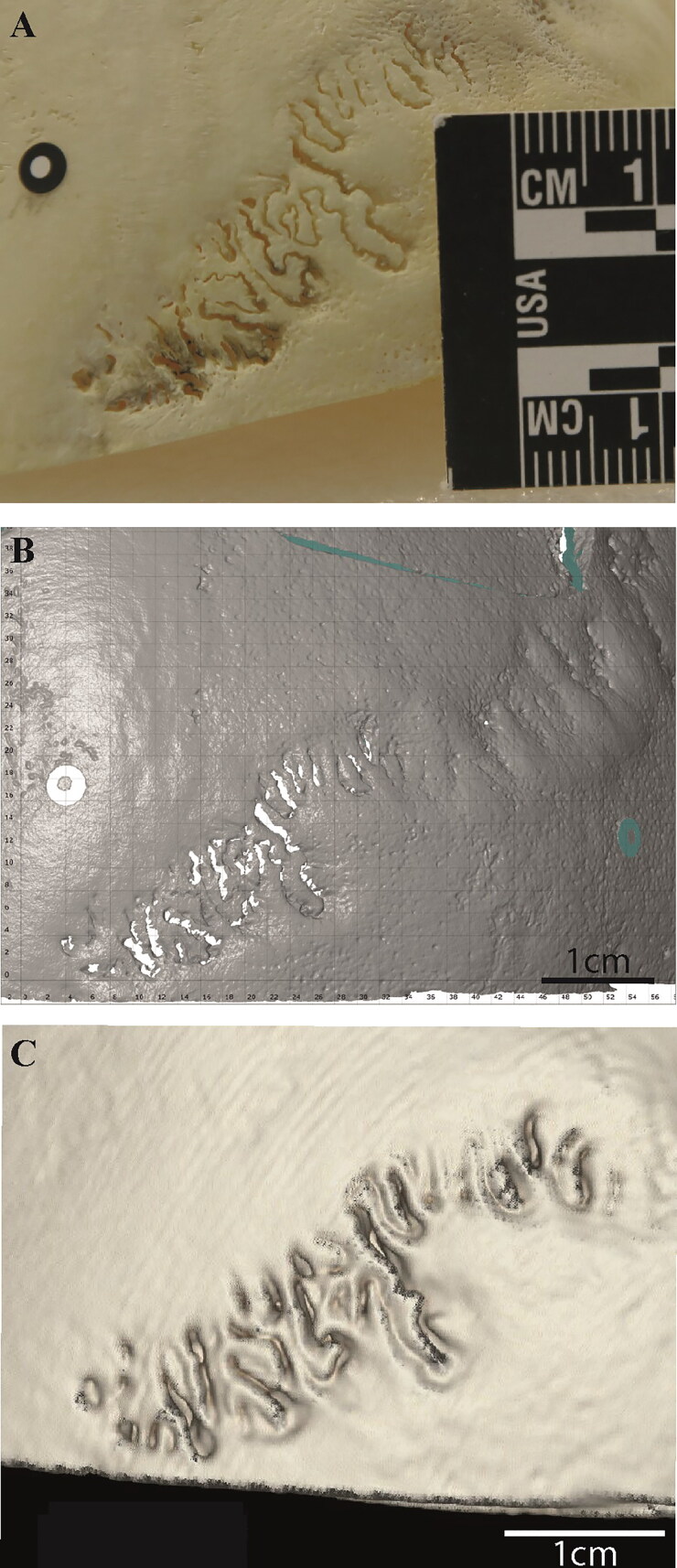
Comparison between macroscopic (A), 3D surface scanning (B), and CT model (C) of a left midlamboid suture. In the CT model (C), the edges of the sutures appear closer together, “rounded”, and less complex than in the dry bone observation. This image demonstrates the shift in perception between three observation media. In A and C, the scale is in cm; in B, it is in mm.

In terms of the pairwise comparison of methods, the results were edifying. It appears that whilst macroscopic observations remain constant, the data collected from 3D surface scans and CT scans differ from one another. Moreover, it appears that observations made on digital models from 3D surface scans differed from macroscopic observation of the skulls (Figure 4). CT models, however, prove reliable. There were no statistically significant differences between the results obtained when comparing macroscopic observations to CT scan observations, whether the data were that of Observer 1 (*P* = 0.6886), Observer 2 (*P* = 0.0940), or the average of both observers (*P* = 0.2099). It appears therefore that while 3D surface scans remain problematic, CT surface scans offer a reliable observation medium. Several observations might help comprehend this phenomenon.

Zooming in when observing a bone macroscopically is, by definition, limited to the observer’s eyesight. Hence, it is easier with this method to limit oneself to the prescribed 1 cm area around the observed cranial suture point. The observer’s vision is similarly limited by the fact that the skull can only safely be held in a number of ways. On 3D surface scans and CT scans, however, the observer can zoom in and out much more extensively and manipulate the skull into viewing angles that would otherwise not be attainable. This can induce biases in suture closure assessment: focalising on a smaller region than originally planned or perceiving a suture as open or closed due to the light reflection on a particular viewing plane.

3D surface scanning models do tend to interpret sutures as either “filled in” or “emptied”. However, as sutures close gradually, this misinterpretation can lead to some sutures appearing completely open when in fact they have started ossifying. This is clearly visible in Figure 4, where the left midlamboid suture is shown as a photograph (Figure 4A), from a 3D surface scan perspective (Figure 4B), and a CT-scan (Figure 4C). The comparison of the CT model with the two others is striking. In that case, the sutures appear further ossified, but also less complex, more “rounded” than in the original dry bone. Meanwhile, from the 3D surface scan point of view, the sutures appear very open and the complex bone bridges between suture edges less defined. These problems with the 3D models from 3D surface scans could be due to the chosen measuring volume of 150 mm × 110 mm × 110 mm, which leads to a resolution of 0.062 mm point distance. This is perhaps not high enough for the aim of this study. By calibrating the scanner with a smaller measuring volume or using another high-resolution surface scanner which leads to a higher model resolution, the analysis of 3D surface scan data will perhaps come closer to the macroscopic observations.

These observations contribute to our understanding of why some models and methods have to be tested and evaluated before they become standard procedures in VA. This observation is even more important considering the nature of the anatomical landmarks investigated here: sutures. These need to be interpreted by the software as “open” or “close”, which might be difficult. In the future, closed anatomical reliefs such as the pubic symphysis or the auricular surface of the pelvis could make better candidates for this type of research.

Most VA research aims to test a specific method (is this method reliable on 3D models). This has been the case in studies looking at cranial suture closure in virtual models, with varying degrees of success [25–27]. Their results, however, are not comparable to those presented in this article. First, because the methodology applied is sometimes different from the original one (choosing to consider cross section through the suture to visualise both endo- and exocranial plates), and second because these studies aimed to evaluate the *accuracy* of the method in a new observation medium rather than the differences in the way the observations are made. In this research, the aim was solely to evaluate if the observation medium (macroscopic observation, 3D surface scans, CT scans) made a difference in how the observer perceived the sutures. This approach is in line with other studies that have attempted to quantify potential differences induced by observation media, although these tend to focus on measurement-based methodologies rather than those relying on descriptive criteria [31, 32], despite the fact that these descriptive criteria are largely used in current anthropological investigations.

In the future, the topic should generate further research, including comparing the results of qualified *versus* quantified methods when transposed from dry bone to 3D models. Furthermore, many studies appear to focus on either the skull [1, 25, 27] or the pelvis [4–6]. This is logical as these skeletal elements are those most used for age and sex determination purposes in adults. Ideally, other postcranial elements such as long bones should be included, and methodologies regarding infants and children should be developed. Altogether, this preliminary research is a promising step towards the systematic evaluation of the suitability of transposing anthropologist’s usual observation methods to computer-generated 3D models. It shows that as far as sutures are concerned, CT scans offer an observation that is closer to the macroscopic view of the original skull than 3D surface scans. This opens up new lines of enquiry into 3D surface scan and CT scan application, as well as the kind of structures and methods that could be most suitable for anthropological analysis in a virtual environment.

This research questions whether methods developed for use on dry bone are readily transferrable to 3D models. As seen through this study, methods developed based on macroscopic observations can yield very different results when applied to digital 3D models. In the case of this research, which focused on a complex anatomical landmark which the software needed to depict correctly in order to interpret if the sutures are open or close, 3D surface scans performed poorly while data from CT scans were comparable to macroscopic observation. These results show that as the use of digital models become commonplace in anthropology, it is crucial that each method routinely used in anthropological research is tested for its reliability when transposed to the digital environment.

## Supplementary Material

Supplemental Material
